# Functional and sequence-based comparison of *Ctenocephalides felis* and *Rhipicephalus sanguineus* sensu lato isolates from different geographic regions

**DOI:** 10.1186/s13071-025-06806-y

**Published:** 2025-05-12

**Authors:** Carolin Schneider, Heike Williams, Hartmut Zoller, Margaret Werr, Claudia Plehn, Eva Zschiesche, Lea Heinau

**Affiliations:** MSD Animal Health Innovation GmbH, Zur Propstei, 55270 Schwabenheim an der Selz, Germany

**Keywords:** *Ctenocephalides felis*, *Rhipicephalus sanguineus*, Fluralaner, GABAR, *Rdl*, Glutamate-gated chloride channel

## Abstract

**Background:**

Current regulatory requirements for European marketing authorization of canine and feline ectoparasiticides include dose confirmation studies conducted with European isolates of each ectoparasite species indicated in the proposed labeling, in addition to any studies conducted against non-European ectoparasite isolates. This regulatory requirement may be deemed unnecessary if no significant differences exist among laboratory ectoparasite isolates obtained from various countries. Between-country interchangeability was examined through various comparative studies by using *Ctenocephalides felis* fleas and *Rhipicephalus sanguineus* sensu lato ticks sourced from multiple countries. To detect potential alterations that may influence fluralaner binding efficacy, comparative complementary DNA (cDNA) and genomic DNA sequence analyses were performed. These analyses focused on the regions coding for the amino acid residues involved in fluralaner binding in the γ-aminobutyric acid receptor (GABAR, subunits encoded by *Rdl*) and the glutamate-gated chloride channel (GluCl) of the mentioned parasite isolates. Additionally, their in vitro fluralaner sensitivities were compared.

**Methods:**

Laboratory in vivo-reared *C. felis* and *R. sanguineus* isolates were sourced from Australia (fleas only), Europe, and USA (both fleas and ticks). Genomic DNA and cDNA sequences coding for GABAR and GluCl were analyzed for variations that could result in alterations of fluralaner and dieldrin binding. For in vitro testing, three replicates of 20 fleas per isolate were exposed to fluralaner-impregnated filter paper at increasing concentrations. In total, three replicates of ten ticks per concentration were immersed for approximately 5 min in fluralaner dilutions. Untreated control replicates were included for all comparisons. Intraclass correlation coefficients were calculated to assess the overall similarity of tick/flea mortality and inhibition rates between different isolates.

**Results:**

No mutation or alteration that could affect the GABAR or GluCl isoxazoline binding efficacy was found in any of three *C. felis* or two *R. sanguineus* isolates. The predicted lethal and effective concentrations of all tested isolates fell within a narrow range. High intraclass correlation coefficients confirmed an overall similarity between tick (Europe and USA) and flea isolates (Europe, USA, Australia) in the in vitro fluralaner sensitivity assay, aligning with the expected susceptibility based on the DNA sequence analysis of each isolate.

**Conclusions:**

There was no evidence of target-related or functional differences in fluralaner sensitivity between either *C. felis* or *R. sanguineus* laboratory isolates from different countries. These findings indicate the between-country interchangeability of results of in vivo dose confirmation studies and justify reducing the number of in vivo studies required for European marketing authorization of canine and feline ectoparasiticides.

**Graphical Abstract:**

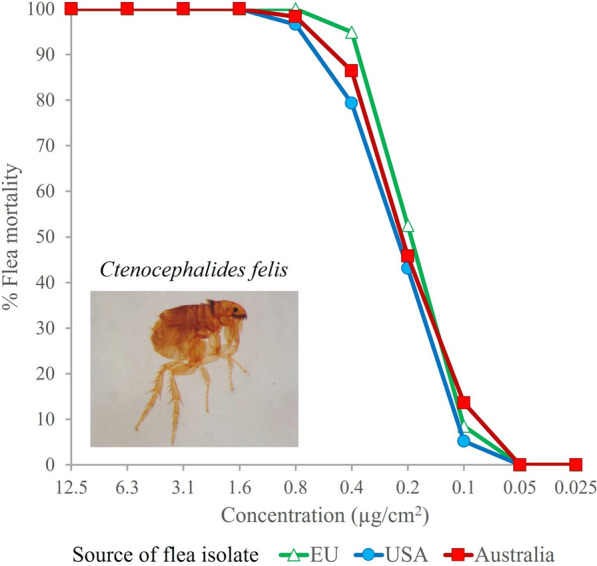

**Supplementary Information:**

The online version contains supplementary material available at 10.1186/s13071-025-06806-y.

## Background

Requirements of the European Medicine Agency (EMA) to demonstrate the efficacy of ectoparasiticides include two dose confirmation studies (at least 12 animals in each study) involving experimental infestations of target hosts using a parasite isolate considered to be representative of the European field situation [1]. Thus, even though laboratory efficacy may have been demonstrated, for instance, against a US isolate of a globally occurring parasite species (such as *Rhipicephalus sanguineus* sensu lato and* Ctenocephalides felis*), additional laboratory testing is a regulatory requirement to validate results against a European isolate for successful registration in the European Union.

This EMA requirement has recently been applied to compounds within the chemical class of isoxazolines. A long-acting member of that chemical class, fluralaner, is now used throughout the world in oral, topical, and injectable formulations to control a range of ectoparasites, including tick and flea infestations of dogs and cats [2, 3]. As with other isoxazolines, fluralaner’s primary targets are binding sites on the insect and acarine γ-aminobutyric acid receptor (GABAR), with a lesser affinity for the glutamate-gated chloride channel (GluCl) [4–7].

A combined in vitro study setup was designed to reduce the number of animal studies in the registration process following the animal welfare principles of replacement, reduction, and refinement (3Rs) [8, 9]. Using laboratory isolates of* C. felis* from Europe, the USA, and Australia, as well as *R. sanguineus* sensu lato (s.l.) from laboratories in Europe and the USA, the project employed two integrated approaches. The first approach aimed to identify any variations in complementary DNA (cDNA) or DNA coding for the amino acid residues involved in fluralaner binding [6, 10–12] in the γ-aminobutyric acid receptor (GABAR) and the glutamate-gated chloride channel (GluCl) among the mentioned parasite isolates. The second approach aimed to determine if there were relevant differences in the sensitivity of these flea and tick isolates to different concentrations of fluralaner.

If the resulting in vitro data show that no relevant differences exist between flea (*C. felis*) and ticks (*R. sanguineus* s.l.) from the USA, Australia, and Europe, additional in vivo laboratory testing of a European isolate in addition to existing in vivo efficacy data of the non-European isolates could not be indicated.

## Methods

### Fleas and ticks

Laboratory in vivo-reared isolates of *R. sanguineus* s.l. and *C. felis* from different continents were sourced for the studies. The European source of *C. felis* originated with a 2013 field collection in Dieburg, Germany that was maintained in the laboratory of MSD Animal Health (Germany) in an in vivo-bred colony, with enrichment in 2019 from wild-type fleas collected from Hungary. The US isolate was originally sourced from Professional Laboratory and Research Services, Inc. (PLRS) in North Carolina, enriched with fleas from Sierra Research in California, USA in 2017, and maintained and supplied for the study from the in vivo-bred colony of Clinvet, South Africa. The Australian isolate originated in 2015 from a household in Scarborough, Western Australia; it was maintained at Von Berky Veterinary Services until 2018 when it was transferred to Eurofins Animal Health and was enriched in 2020 with fleas collected from the field in New South Wales. The colony was maintained on dogs at Eurofins. Resistance status for the Europe and USA isolates was not defined, while bioassays of the Australian isolate showed a high resistance to fipronil.

The European *R. sanguineus* s.l. isolate was originally sourced by Charles River Laboratories from Oxford (United Kingdom) in 2002. To maintain genetic diversity, additional *R. sanguineus* s.l. ticks were introduced from Germany and Spain in May 2007 and May 2009, respectively. The US isolate originated from a field collection in Bulloch County, Georgia, and was maintained for at least one generation at Utrecht University, Netherlands. In 2014, a colony derived from this isolate was initiated at Clinvet South Africa, which provided ticks to be used in the study. Ticks were morphologically identified only and are referred to herein as *R. sanguineus* s.l., as discussed elsewhere [13].

### Comparative sequence analysis, GABAR, and GluCl

#### Ctenocephalides felis

To isolate DNA using a genome-based approach, 20 fleas from each strain were placed into a tube, cooled to reduce mobility, placed in a dish with sterile, distilled water, and then pipetted individually (i.e., one flea/vial) into Precellys^®^ tubes (Precellys^®^ ceramic kit 2.8 mm; pEQlab) containing 500 µl TRIzol (Ambion). The fleas were then homogenized twice, with a 10-min separation at 45 s, 6500 rpm (Precellys^®^ homogenizer, Bertin Instruments, Montigny-le-Bretonneux, France). The supernatant was transferred into an Eppendorf tube, and genomic DNA precipitated by adding 1000 µl ethanol and incubating on ice for 10 min. After centrifugation, the DNA pellet was washed with ice-cold 70% ethanol and centrifuged again. The supernatant was removed, and the DNA pellets were dried and resuspended in 100 µl Tris-Buffer. The DNA was stored at 4 °C.

To amplify the sequences of exon 6 of the *C. felis* GABA receptor subunit gene (*Rdl*), 2 µl of the DNA probes from each isolate were used as templates. For each strain, 12–15 polymerase chain reaction (PCR) reactions were performed with the individual genomic DNA, using 35 cycles with primer CfGABAR-Resist-for 5′GGAAACTATTCTCGCCTGGCGTGC and CfGABAR-Resist-rev1 5′CAA TCAACTTACCTAATAAACTAGC. The primers were designed using the *C. felis* whole genome shotgun sequence with National Center for Biotechnology Information (NCBI) Reference Sequence: NW_020538040.1 as a template. The reverse primer covers the exon 6-intron boundary of the *Rdl* gene.

To amplify exon 6 of the *C. felis* GluCl gene, DNA probes were used as templates. For each strain, 16 PCR reactions with the individual genomic DNA were performed using 40 cycles (1 × 94 °C for 180 s; 40 × 94 °C for 30 s, 56 °C for 30 s, 72 °C for 40 s; 1 × 72 °C for 300 s; 4 °C) using primer combination as described below. The primers (Cf-GluCl-Exon6-for 5′GTGTGACCACTCTCCTCACCATGG 3′ and Cf-GluCl-Exon6-rev 5′CATAGCGAA GGTACCATCAGTGC 3′ were designed using the *C. felis* whole genome shotgun sequence (NCBI Reference Sequence: XM_026624636.1|:728–999) as template. After amplification, the probes were analyzed and purified by agarose gel electrophoresis. Fragments of interest were excised and purified using the Gene Clean Kit II (MP Biomedicals, California, USA). The purified PCR fragments were diluted, 12 fragments from each strain were sent to GATC (Eurofins) for sequencing. The sequences were translated and aligned using DNAStar-MegAlign (ClustalW). Annotations of the transmembrane domain, the predicted fluralaner, and dieldrin resistance binding sites were compared with previously reported sites [6, 10–12]. From all sequenced PCR fragments, 33 *Rdl* gene sequences of exon 6 (11 from each isolate) were obtained. Sequences with gaps, poor quality, or unspecific sequences were excluded from further analysis.

### *Rhipicephalus sanguineus* s.l.

The cDNA of male and female unfed adult *R. sanguineus* s.l. ticks was generated by using the SMARTer^®^ RACE 5′/3′ (Takara Bio, USA). For first-strand cDNA synthesis, total RNA was primed using a modified oligo (dT) primer. For amplification of the full-length RsGABAR and RsGluCl sequences, the 5′RACE cDNA probes (European or USA isolate cDNA) were used as templates. The PCR reaction was performed using 40 cycles with high-fidelity DNA polymerase using the following primer combinations: (BmGabaR-8.1-Full-for 5′ATGAGACAAGCGATGGCGTTCAGTTG 3′ plus

BmGabaR-8.1-Full-rev 5′CTAGTCGTCGCCGACATCGTCC3′ or BmGABAR-Full-for 5′ GGATCCACCATGAGACAAGCGATGGCGTTCAGT plus BmGABAR-Full-rev 5’TGC

TGCGGCCGCCTAGTCGTCGCCGACATCGTCCGGCAGAACG) or GluCl (RsGluCl-spez-for2 5′ACCGACTACTGCACCAGTCG3′ plus RsGluCl-spez-rev1 5′AATCTGACCGCG AGGCGTAG 3′) as template. After amplification, the probes were analyzed by agarose gel electrophoresis, cloned, and sequenced. From all sequenced clones, we obtained 16 full-length *Rdl* (GABAR) sequences (8 from each strain) and 22 *GluCl* sequences (12 and 10 from Europe and USA isolates, respectively). Sequences with gaps, poor quality, or unspecific sequences were excluded from further analysis. The sequences were translated and analyzed by alignment with protein sequences of *Drosophila melanogaster* and *Musca domestica* (*D.melanogaster* Rdl isoformA, accession no.: NP_523991.2, *M. domestica* GABAR subunit, accession no.: BAD16658.2, and *M. domestica* glutamate-gated chloride channel subunit type A, accession no.: BAD16657.1). The alignment with those subunits allowed for the annotation of key sites known to confer interaction or resistance [6, 10–12].

### Fluralaner contact sensitivity

Test solutions were prepared by adding deionized water to a solution of fluralaner in dimethyl sulfoxide/emulsifier mixture to obtain fluralaner test concentrations. For all contact sensitivity tests, a dimethyl sulfoxide premix solution with a solvent concentration of either 2% (flea) or 5% (tick) equivalent to the premix concentration in the highest concentrated fluralaner test solution per test (tick/flea) served as a solvent control. Additionally, a second control group was left unexposed to any test solutions for each test.

#### Ctenocephalides felis

For contact exposure assessments, fluralaner test concentrations ranged from 1000 to 2 ppm (equivalent to 12.5 to 0.025 μg/cm^2^). A total of three replicates each of 20 fleas per isolate per test concentration (in total, 60 fleas per isolate, per concentration) were placed into glass vials containing moist fluralaner-impregnated filter paper strips. Vials were closed with an air-permeable lid and incubated at approximately 22 °C and 80% relative humidity. After approximately 48 h of contact exposure, fleas in each vial were visually assessed under a magnifying glass. Those not showing any movement after stimulation (e.g., by exhaling into a vial through the air-permeable lid) were assessed as dead, and those with trembling legs, unable to right, or appearing uncoordinated were assessed as damaged. Live, damaged, and dead fleas were counted.

### *Rhipicephalus sanguineus* s.l.

For the contact exposure study, unfed adult ticks were used. Dilutions for immersion testing ranged from 2000 to 1 ppm (equivalent to 2000 μg/ml to 1 μg/ml). Using a warm surface to stimulate activity, ticks were sorted from their colonies, sexed, and distributed into test tubes (20 ml polypropylene-vials with permeable lids). The next day, ticks were immersed for approximately 5 min in an Erlenmeyer flask containing the fluralaner test concentrations. In total, 30 unfed adults of each isolate were then removed from the flask, collected in a strainer, and dried with toweling paper. From each test concentration, three sets of ticks from each isolate were transferred into Petri dishes lined with dry filter paper (7 cm diameter) to provide three replicates of ten ticks, five male and five female, per isolate per concentration. Petri dishes were covered with a lid, wrapped with Parafilm^®^, and incubated at approximately 28 °C and 85% relative humidity for approximately 48 h. After removal from incubation, ticks were placed onto a heated plate (approximately 40 °C). Those not showing any movement after stimulation (e.g., by exhaling onto ticks and/or touching with a fine brush) were assessed as dead. Ticks with trembling legs or unable to right or show coordinated movement were assessed as damaged. Live, damaged, and dead ticks were counted.

### Analysis

Flea and tick mortalities (%) were calculated for each isolate and fluralaner test concentration using the formula:

$$\left( \% \right){\text{ Mortality}} = \frac{{{\text{D}}_{{\text{T}}} {-}{\text{ D}}_{{\text{C}}} }}{{{\text{R }}{-}{\text{ D}}_{{\text{C}}} }} \times 100$$

where D_C_ is the arithmetic mean (all replicates) of dead fleas/ticks including both negative controls (solvent and untreated control), D_T_ is the total (all replicates) of dead fleas/ticks per concentration of fluralaner, and R is the number of fleas or ticks included at each tested concentration (60 fleas, 30 ticks).

Flea and tick inhibitions (% dead and damaged) were calculated for each isolate and fluralaner test concentration using the following formula:

$$\left( \% \right){\text{ Inhibition}} = \frac{{\left( {{\text{D}}_{{\text{T}}} + {\text{ Da}}_{{\text{T}}} } \right) - \left( {{\text{D}}_{{\text{C}}} + {\text{ Da}}_{{\text{C}}} } \right)}}{{{\text{R }}{-}\left( {{\text{D}}_{{\text{C}}} {-}{\text{ Da}}_{{\text{C}}} } \right)}} \times 100$$

where D_C_ + Da_C_ is the arithmetic mean (all replicates) of dead (D) and damaged (Da) ticks or fleas in the control groups (solvent and untreated control) and D_T_ + Da_T_ are the total number (all replicates) of dead (D) and damaged ticks or fleas (Da) per concentration of fluralaner, and R is the number of ticks or fleas included at each tested concentration (60 fleas or 30 ticks). Lethal concentrations (LC) and effective concentrations (EC) (dead and damaged fleas or ticks) for 50% (EC_50_) and 90% (EC_90_) were calculated for each isolate using Excel (XL fit, release 5.3.1.3; 2006‒2011).

### Statistical comparison of tick/flea mortality and inhibition rates of different isolates

Intraclass correlation coefficients (ICC), according to Shrout–Fleiss (1979) were determined to assess overall similarity of tick/flea mortality and inhibition rates between different laboratory isolates [14].

- ICC (1,1): single score intraclass correlation coefficient, without bias
- ICC (2,1): random set, absolute agreement ICC score in the presence of bias
- ICC (3,1): fixed set, consistency ICC score in the presence of bias

Intraclass correlation coefficients are measures between 0 and 1, where higher values denote increasing similarity between the compared sets.

As concluded by Liljequist et al. [15], “all three single-score intraclass correlation coefficients may be calculated and compared.” Both ICC (2,1) and ICC (3,1) are “valid in the presence as well as in the absence of measurement bias.”

## Results and discussion

### Comparative sequence analysis, GABAR and GluCl

Fluralaner is a potent inhibitor of the ionotropic GABAR, with a less potent, but nonetheless significant, inhibition of GluCl [5]. The binding of fluralaner to the GABAR and subunit binding sites have been well-reported [6, 10–12]. The isoxazoline GABAR binding pocket is described as located in the subunit interface of TM1 and TM3 [10]. The positions Q271, I274, and L278 in the TM1 as well as G333 in TM3 were found to be the most important sites for sensitivity to fluralaner; an alteration in position Q271, L278, and G333 leads to a decrease of the half maximal inhibitory concentration (IC_50_) value and alteration in position I274 an increase. The importance of position G333 in TM3 could even be demonstrated in vivo by CRIPR/Cas9-generated *Drosophila melanogaster* mutants [11]. Homozygous *D. melanogaster* lines carrying the G/M mutation in this position showed high resistance to fluralaner and broflanilide. Additionally, it was recently demonstrated in vitro that mutations in position N316 confer strong resistance to fluralaner [12].

**Fig. 1:**
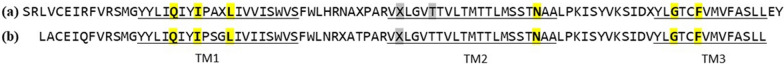
Consensus sequence of multi-way protein sequence alignment of translated GABAR sequences (**a**) *Rhipicephalus sanguineus* s.l. Europe and USA, and (**b**) *Ctenocephalides felis* Australia, Europe, and USA. The transmembrane regions 1–3 are underlined. The important fluralaner interaction amino acids are highlighted in yellow, and the dieldrin resistance site is highlighted in grey

No sequence alterations affecting the interaction sites of fluralaner, as described by Yamato et al. [10] and Asahi et al. [12], were found in the *Rdl* gene of *C. felis* (Fig. 1; Additional file 2: Supplementary Fig. S2). The general ability of these study methods to identify resistance is demonstrated by the identification of the dieldrin resistance mutation, which was homozygous in approximately 50% of the investigated US and European flea strains (Additional file 2: Supplementary Fig. S2: Position 2 A/S in TM2, equivalent to position 302 in *D. melanogaster* [17]); heterozygous fleas were detected in both laboratory isolates (a double peak in the sequence chromatogram leads to an ‘N’ in the sequence, indicated as ‘X’ in the translated sequence). The Australian isolate was homozygous for the dieldrin resistance mutation.

Similarly, in the *R. sanguineus* s.l. laboratory isolates, no alterations were observed in any sequence coding for the key fluralaner GABAR interaction amino acid residues, compared with the reference sequences (Fig. 1; Additional file 1: Supplementary Fig S1). A dieldrin resistance site in position 6 of TM2 (Fig. 1, position 303) was found in both isolates [18]. Additionally, consistent with an earlier report [19], an AS/TA exchange was detected at TM3. While the significance of this exchange is not clear, it may result in a modification of the receptor’s three-dimensional structure that is associated with fipronil resistance [19].

Fluralaner also has an antagonistic action on the GluCl, the main target of macrocyclic lactone parasiticides [5]. Although the GluCl is not the main target for fluralaner, we also tested for possible sequence alterations in the *R. sanguineus* s.l. and *C. felis* isolates.

**Fig. 2:**
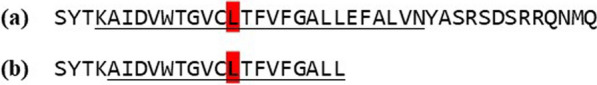
Consensus sequence of multi-way protein sequence alignment of translated GluCl sequences of (**a**) *Rhipicephalus sanguineus* s.l. USA and CRL (Europe) and (**b**) *Ctenocephalides felis* GluCl sequences Europe, USA, and Australia (annotated by alignment with MdGluCl (BAD16657). The important fluralaner interaction amino acid leucine 315 (L315) is highlighted in red. No differences in position L315 are observed. The transmembrane region (TM) is underlined

In *C. felis*, the position coding for the putative fluralaner GluCl interaction site, important for the selectivity of both fluralaner and ivermectin, is located in exon 6 of the GluCl gene (corresponds to Leucin 315 in TM3 from *M. domestica* sequence) [16]. None of the *C. felis* GluCl sequences of exon 6 (11 from each isolate) revealed alterations in any of the investigated flea isolates coding for this important position when compared with the sequences described by Nakata et al. 2017 (Fig. 2). All tested fleas were homozygous in this position (Additional file 3: Supplementary Fig. S3).

All sequenced *R. sanguineus* s.l. fragments, in total 22 GluCl sequences (12 and 10 from Europe and USA isolates, respectively), were analyzed, translated, and annotated by alignment with *M. domestica* GluCl subunit type A (Fig. 2; Additional file 3: Supplementary Fig. S3). The annotation and comparison of all sequences reveal no differences in the predicted fluralaner interaction site of the GluCl. The sequences showed an outmost high degree of conservation.

No site exchanges were detected exclusively throughout any *R. sanguineus* s.l. isolate. The detected single-site exchanges (e.g., Y538 C) are most likely nonsynonymous substitution SNPs or a result of the cDNA synthesis or PCR reaction. Since there were no protein sequence differences between the two isolates at the known fluralaner interaction sites, it can be assumed that no on-target related sensitivity differences were present in either *R. sanguineus* s.l. isolate.

### Fluralaner contact sensitivity

**Fig. 3:**
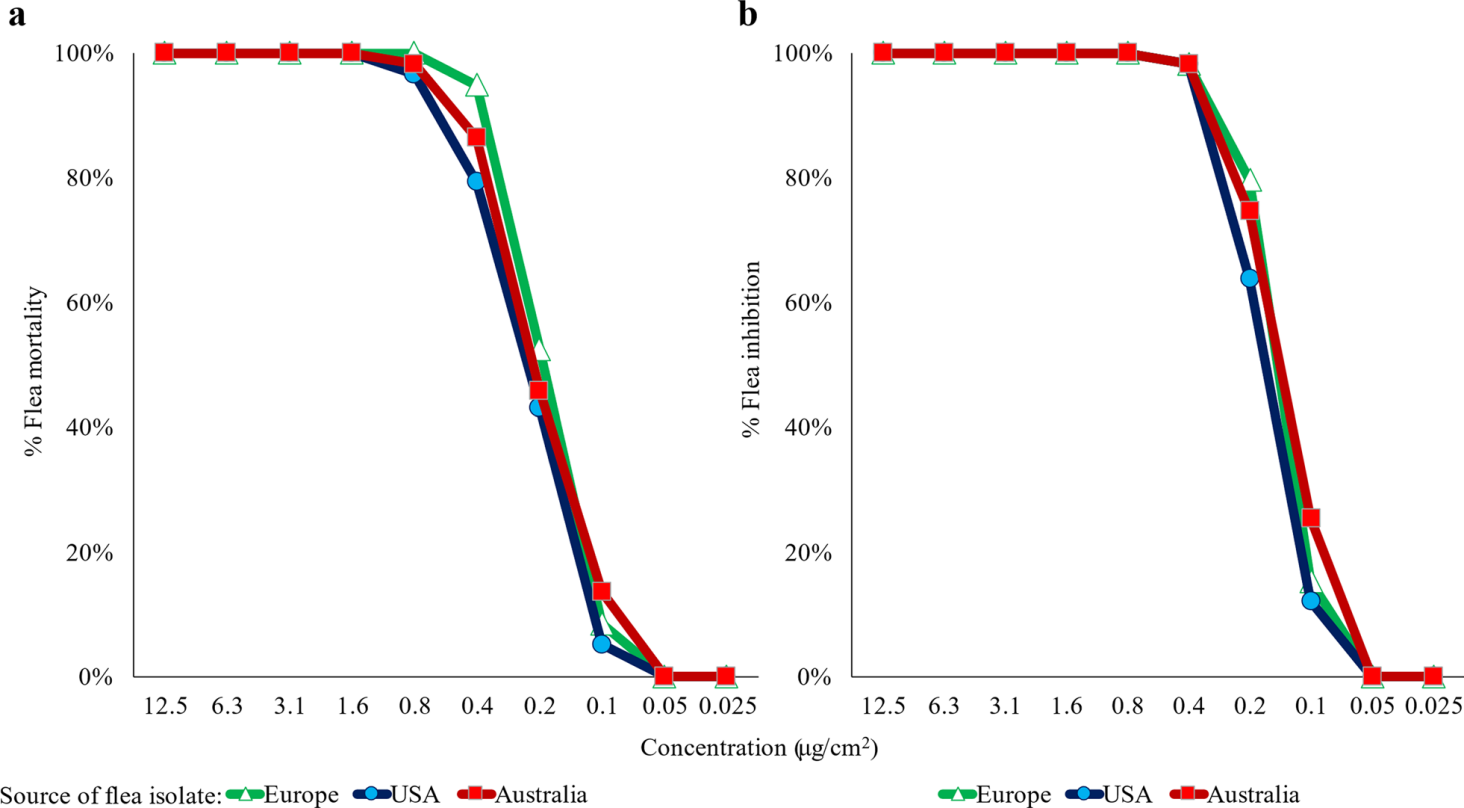
**a** Mortality (dead) and **b** inhibition (dead and damaged) (%) of *Ctenocephalides felis* isolates of different geographic origins after approximately 48 h of contact exposure to fluralaner

Fluralaner demonstrated high in vitro insecticidal contact activity against all three tested laboratory *C. felis* isolates. The predicted lethal and effective concentrations of the different isolates fell within a narrow range (Table 1; Fig. 3). Assessments of live fleas in both negative control groups confirmed the viability of fleas used in the assay.

**Table 1:** Predicted lethal (LC) and effective concentrations (EC) (95% fiducial limits) for *Ctenocephalides felis* isolates after approximately 48 h of fluralaner contact exposure (μg/cm^2^)

Isolate source	LC_50_	EC_50_
Europe	0.15 (0.15, 0.15)	0.15 (0.15, 0.15)
USA	0.17 (0.17, 0.18	0.17 (0.17, 0.18)
Australia	0.14 (0.14, 0.15)	0.14 (0.14, 0.15)

**Fig. 4:**
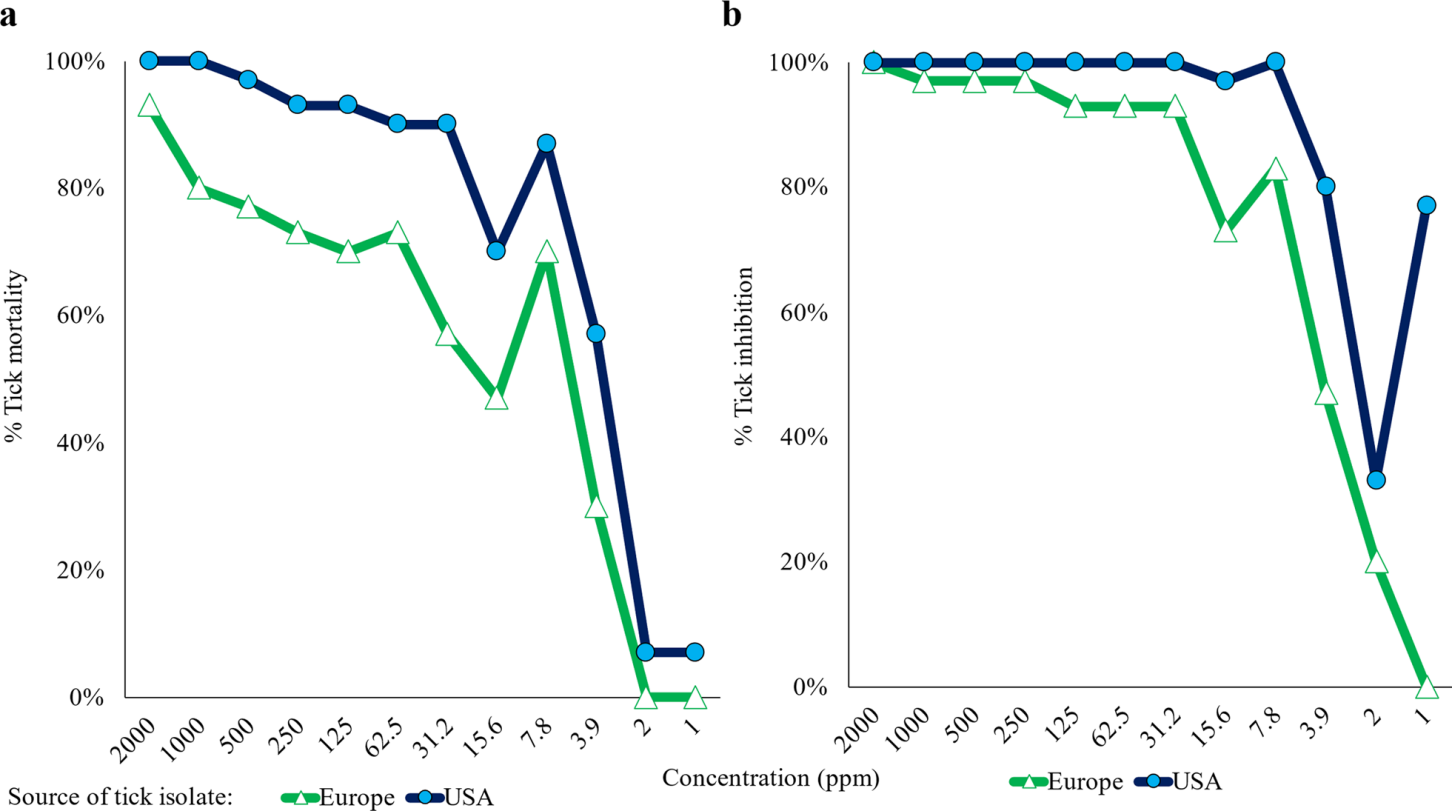
**a** Mortality (dead) and **b** inhibition (dead and damaged) (%) of *Rhipicephalus sanguineus* s.l. isolates of different geographic origins after approximately 48 h of contact exposure to fluralaner

Predicted effective concentration values for the *R. sanguineus* s.l. European isolate were in a comparable range to those for the USA isolate (Table 2; Fig. 4). After short-term contact exposure by immersion, fluralaner demonstrated similar in vitro acaricidal contact activity against both tested laboratory *R. sanguineus* s.l. isolates. Assessments of live ticks in both negative control groups confirmed the viability of ticks used in the assay.

**Table 2:** Predicted effective concentrations (95% fiducial limits) of fluralaner against two *Rhipicephalus sanguineus* sensu lato isolates after fluralaner contact exposure (assessed approximately 48 h after a 5 min immersion) (parts per million)

Isolate source	EC50	LC50
Europe	4.58 (3.13, 6.04)	18.60 (0.78, 36.42)
USA	2.41 (2.18, 2.64)	4.10 (2.65, 5.54)

### Overall similarity of tick/flea mortality and inhibition rates between different isolates

For dead tick counts, ICC scores were as follows: ICC (1,1): 0.83205, ICC (2,1): 0.84300, and ICC (3,1): 0.96945. For dead and damaged tick counts, ICC scores were as follows: ICC (1,1): 0.88543, ICC (2,1): 0.88910, and ICC (3,1): 0.95008.

For both dead and dead and damaged ticks, ICC (3,1) is larger than ICC (2,1), suggesting the presence of some bias. However, for both dead and dead and damaged ticks, all three ICC scores are well above 0.8, indicating an overall high similarity between the tested laboratory tick isolates (Europe and USA), independent from any considerations of measurement bias.

For dead flea counts, ICC scores were as follows: ICC (1,1): 0.99551, ICC (2,1): 0.99551, and ICC (3,1): 0.99612. For dead and damaged flea counts, ICC scores were as follows: ICC (1,1): 0.99502, ICC (2,1): 0.99502, and ICC (3,1): 0.99519.

For both dead and dead and damaged fleas, ICC (3,1) is slightly larger than ICC (2,1), suggesting the presence of some bias. However, for both dead and dead and damaged fleas, all three ICC scores are above 0.99, indicating an overall very high similarity between the tested laboratory flea isolates (Europe, USA, and Australia), independent from any considerations of measurement bias.

### Limitations

The sensitivity testing by immersion/contact exposure of ticks and fleas does not resemble the mode of exposure in vivo, where the active is ingested with the blood meal. However, for sensitivity comparison purposes between isolates and parasite species, this assay is sufficiently accurate and in the case of fluralaner, the result is backed up by a large amount of in vivo data generated with tick and flea isolates from the different regions in question over the years. Therefore, this in vitro approach is based on extensive datasets of fluralaner’s proven antiparasitic efficacy, which allows for accurate interpretation of the in vitro data. The aim of this publication is to show a potential approach for the incorporation of in vitro studies, in this case, for fluralaner in the authorization procedures. The feasibility of this approach for other compounds or parasite species needs to be carefully evaluated taking into consideration regulatory requirements, available in vivo efficacy data for the compound, and availability of suitable in vitro test systems for parasite sensitivity to the compound in question. By including in vitro studies, the number of animal experiments can be reduced while still obtaining meaningful efficacy data, thus promoting the implementation of the 3Rs principles (replacement, reduction, refinement) in animal testing.

## Conclusions

No mutation or alteration that could affect the GABAR or GluCl isoxazoline binding, leading to target-related resistance, was found in any of three *C. felis* laboratory isolates sourced from Europe, the USA, and Australia or the two *R. sanguineus* s.l. laboratory isolates sourced from the USA and Europe. The functional comparison showing equivalent activity between the flea and tick isolates from each country aligns with the expected or predicted susceptibility based on the DNA sequence analysis of each isolate. Thus, the equivalent functional susceptibility of the three *C. felis* isolates and the equivalent susceptibility of the two *R. sanguineus* s.l. isolates to fluralaner indicate that findings from any one isolate can be applied to the other isolate in in vivo studies. Therefore, these findings demonstrate the between-country interchangeability of results in in vivo dose confirmation studies. In addition to justifying reducing the number of required dose confirmation studies, this comparative approach offers the potential for application to other products and parasites, particularly when the initial conditions regarding the active ingredient are similar.

## Supplementary Information

Additional file 1

## Data Availability

No datasets were generated or analyzed during the current study.

## Abbreviations

GABAR: γ-Amino butyric acid receptor
GluCl: Glutamate-gated chloride channel
Rdl: Resistant to dieldrin subunit of the gamma-aminobutyric acid receptor
D_C_: Dead fleas or ticks in the untreated control group
Da_C_: Damaged fleas or ticks in the untreated control group
Da_T_: Damaged fleas or ticks in treated groups
D_T_: Dead treated fleas or ticks in treated groups
R: The number of ticks or fleas included at each tested fluralaner concentration
