# Supplementary material for: Functional and sequence-based comparison of Ctenocephalides felis and Rhipicephalus sanguineus sensu lato isolates from different geographic regions
Source: Parasit Vectors. 2025 May 12;18:170. doi: 10.1186/s13071-025-06806-y (PMC12070772; doi:10.1186/s13071-025-06806-y)
Supplement: Supplementary file 1 — Additional file 1 [file 13071_2025_6806_MOESM1_ESM.docx]

**Supplementary information**


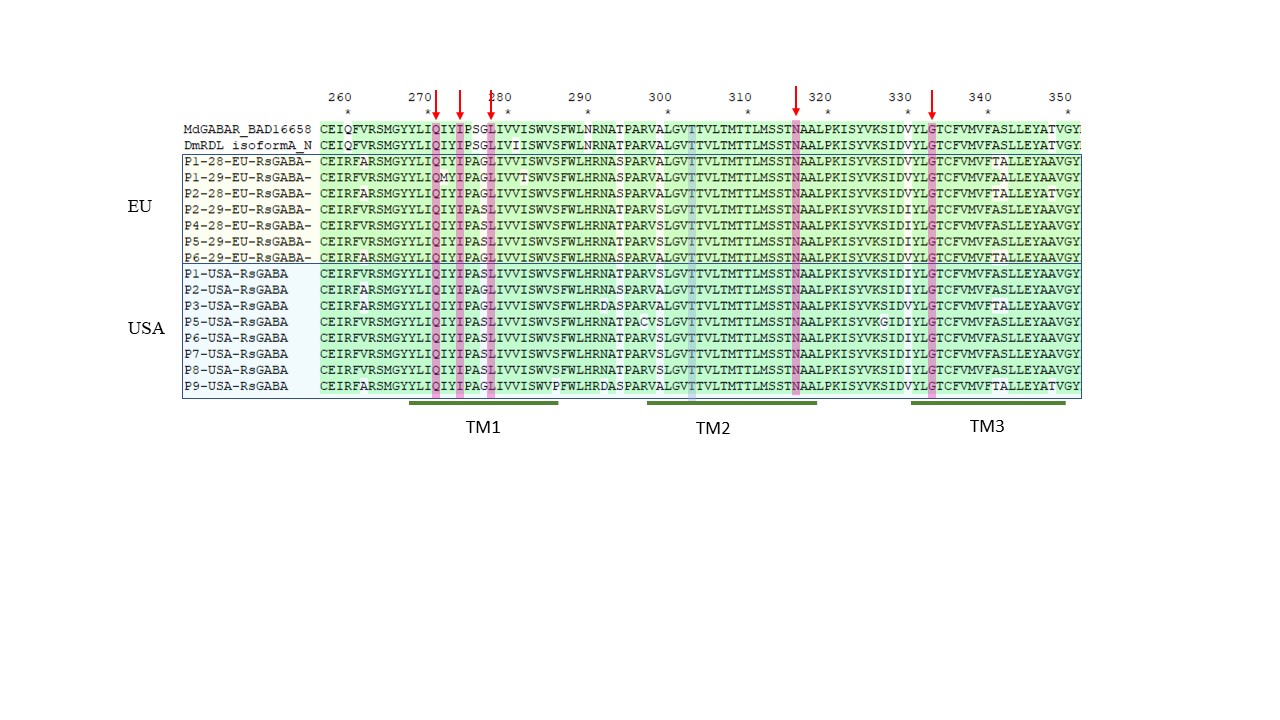


**Additional file 1.** **Fig S1.** Multi-way protein sequence alignment of translated *Rhipicephalus sanguineus* s.l. GABAR sequences USA and Europe (EU) with MdGABAR (BAD16658) and DmRDL. The transmembrane regions 1-3 are indicated by green lines. The important fluralaner interaction amino acids are highlighted in red, and the dieldrin resistance site in grey [18].

**
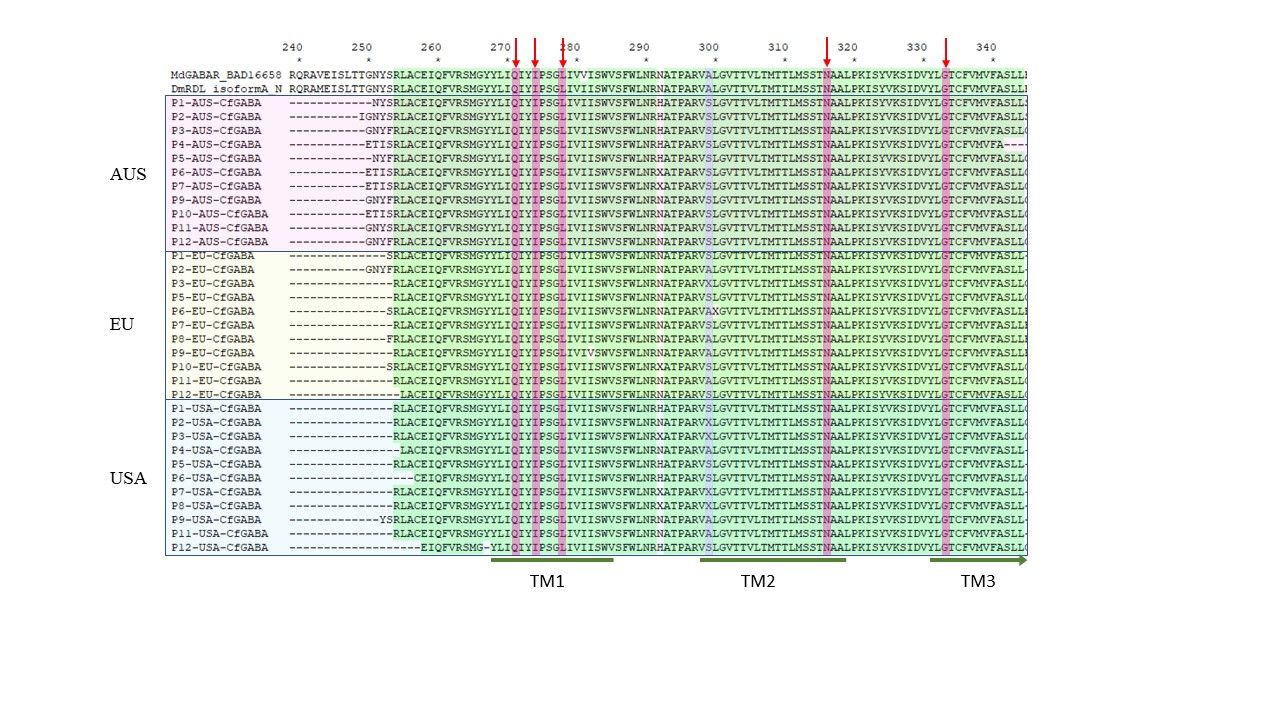
**

**Additional file 2.** **Fig. S2.** Multi-way protein sequence alignment of translated *Ctenocephalides felis* GABAR sequences, Australia (AUS), Europe (EU), and USA with MdGABAR (BAD16658) and DmRDL. The transmembrane regions 1 - 3 are indicated by green lines. The important fluralaner interaction amino acids are highlighted in red, and the dieldrin resistance site in grey.


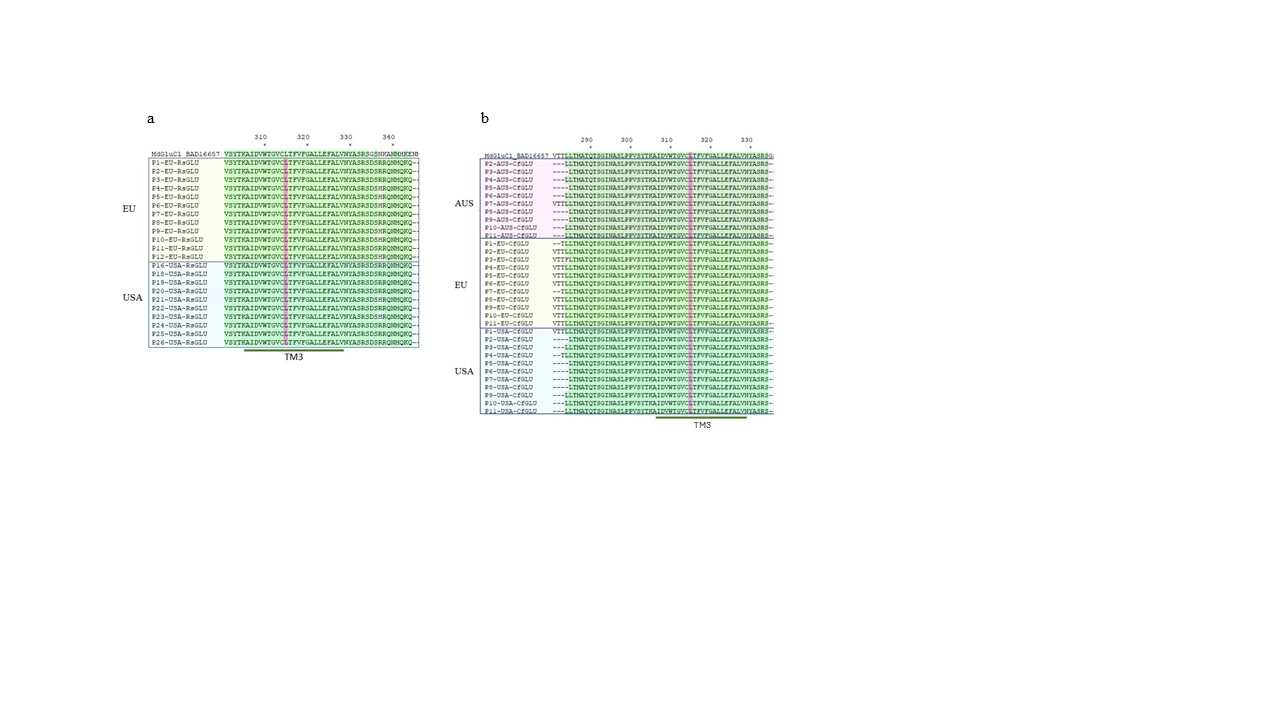


**Additional file 3. Fig S3.** Multi-way protein sequence alignment of translated **a** *Rhipicephalus sanguineus* s.l. GluCl sequences, USA and Europe. No differences in position L315 are observed. The important fluralaner interaction amino acid L315 (leucine 315) is highlighted in red. The transmembrane region (TM3) is indicated by the green line, **b** *Ctenocephalides felis* GluCl sequences Europe, USA, and Australia with MdGluCl (BAD16657). Heterozygous fleas would have been detected by a double peak in the sequence chromatogram. The double peak leads to an 'N' in the sequence which would have been indicated as 'X' in the translated sequence.
